# Induction of Foxp3-Expressing Regulatory T-Cells by Donor Blood Transfusion Is Required for Tolerance to Rat Liver Allografts

**DOI:** 10.1371/journal.pone.0007840

**Published:** 2009-11-23

**Authors:** Yuta Abe, Hidejiro Urakami, Dmitry Ostanin, Gazi Zibari, Tetsu Hayashida, Yuko Kitagawa, Matthew B. Grisham

**Affiliations:** 1 Department of Molecular and Cellular Physiology, Louisiana State University Health Sciences Center, Shreveport, Louisiana, United States of America; 2 Department of Surgery, Louisiana State University Health Sciences Center, Shreveport, Louisiana, United States of America; 3 Department of Surgery, Keio University School of Medicine, Tokyo, Japan; New York University School of Medicine, United States of America

## Abstract

**Background:**

Donor-specific blood transfusion (DST) prior to solid organ transplantation has been shown to induce long-term allograft survival in the absence of immunosuppressive therapy. Although the mechanisms underlying DST-induced allograft tolerance are not well defined, there is evidence to suggest DST induces one or more populations of antigen-specific regulatory cells that suppress allograft rejection. However, neither the identity nor the regulatory properties of these tolerogenic lymphocytes have been reported. Therefore, the objective of this study was to define the kinetics, phenotype and suppressive function of the regulatory cells induced by DST alone or in combination with liver allograft transplantation (LTx).

**Methodology/Principal Findings:**

Tolerance to Dark Agouti (DA; RT1^a^) rat liver allografts was induced by injection (iv) of 1 ml of heparinized DA blood to naïve Lewis (LEW; RT1^l^) rats once per week for 4 weeks prior to LTx. We found that preoperative DST alone generates CD4^+^ T-cells that when transferred into naïve LEW recipients are capable of suppressing DA liver allograft rejection and promoting long-term survival of the graft and recipient. However, these DST-generated T-cells did not express the regulatory T-cell (Treg) transcription factor Foxp3 nor did they suppress alloantigen (DA)-induced activation of LEW T-cells *in vitro* suggesting that these lymphocytes are not fully functional regulatory Tregs. We did observe that DST+LTx (but not DST alone) induced the time-dependent formation of CD4^+^Foxp3^+^ Tregs that potently suppressed alloantigen-induced activation of naïve LEW T-cells *in vitro* and liver allograft rejection *in vivo*. Finally, we present data demonstrating that virtually all of the Foxp3-expressing Tregs reside within the CD4^+^CD45RC^−^ population whereas in which approximately 50% of these Tregs express CD25.

**Conclusions/Significance:**

We conclude that preoperative DST, in the absence of liver allograft transplantation, induces the formation of CD4^+^ T-cells that are not themselves Tregs but give rise directly or indirectly to fully functional CD4^+^CD45RC^−^Foxp3^+^Tregs when transferred into MHC mismatched recipients prior to LTx. These Tregs possess potent suppressive activity and are capable of suppressing acute liver allograft rejection. Understanding the mechanisms by which preoperative DST induces the generation of tolerogenic Tregs in the presence of alloantigens may lead to the development of novel antigen-specific immunological therapies for the treatment of solid organ rejection.

## Introduction

Transplantation of different organs across major histocompatability complex (MHC) barriers induces potent effector responses by the immune system resulting in the substantial tissue injury and rejection of the allograft. Indeed, transplantation of certain organ allografts results in rapid rejection of the allograft in the absence of immunosuppressive therapy. With the advent and long-term use of potent immunosuppressive agents, acute rejection has been substantially reduced and allograft survival has increased dramatically over the past two decades[1]. However, these nonspecific immunosuppressive drugs possess several significant limitations and side effects which may limit their long-term uses[2], [3]. The ideal therapy for inducing long-term tolerance to tisse allografts would be induction of antigen-specific suppression of the acute and chronic rejection without compromising host defenses. Over the past 20 years there have been numerous experimental and clinical reports demonstrating that tolerance can be induced to certain allografts by infusion of different donor cell preparations[4], [5]. Indeed, donor-specific blood transfusion (DST) has been shown to induce immune hyporesponsiveness and tolerance to a number of different tissues including heart, kidney and liver in the absence of exogenous immunosuppressive agents[5]–[12]. It is known for example, that DST significantly prolongs heart or liver allograft survival however mononuclear/lymphocyte cell infiltration into DST-treated allografts was found to be as great (or greater) when compared to untreated grafts in early phase following transplantation[13], [14].These data suggested that deletion of alloreactive T-cells may not be the mechanism by which allografts are protected from acute rejection. In addition, it has been shown that donor MHC positive-cells accumulate in the spleen and lymph nodes at 12 hr following DST and that splenectomy performed at the time of transplantation abrogated tolerance to liver allografts[15], suggesting the importance of secondary lymphoid system for tolerance induction. In addition, expression of the immunoregulatory cytokines TGFβ and IL-4[13] as well as the numbers of CD4^+^CD45RC^−^ T-cells[14] have been reported to be increased in the tolerated allografts suggesting the presence regulatory T-cells (Tregs). Indeed, adoptive transfer of CD4^+^CD45RC^−^ T-cells obtained from rats that received DST and heart allograft into naïve recipients induced long term tolerance to *heart allografts* suggesting the presence of Tregs within this lymphocyte population[16]. However, neither the identity nor the regulatory properties of the Tregs present within the CD4^+^CD45RC^−^ T-cell subset responsible for DST-induced allograft tolerance was investigated. Therefore, the objective of this study was to characterize the kinetics, phenotype and suppressive function of the Tregs induced by DST alone or in combination with liver allograft transplantation (LTx). We report that preoperative DST induces the formation of CD4^+^ T-cells that are not themselves classical Tregs but develop into fully functional Foxp3^+^ Tregs or help to induce the formation of CD4^+^CD45RC^−^Foxp3^+^ Tregs following allograft transplantation.

## Materials and Methods

### Animals

Inbred male MHC-mismatched Dark Agouti (DA; RT1^a^) rats, Lewis (LEW; RT1^l^) rats and Piebald virol Glaxo pigmented rats (PVG; RT1^c^) were purchased from Harlan Laboratories (Indianapolis, IN) and used as donors, recipients and third party donor controls, respectively. All animals were fed normal rat food *ad libitum* with free access to water. The care and use of laboratory animals conformed to the National Institutes of Health and LSUHSC guidelines.

### Orthotopic Liver Transplantation and Donor Specific Blood Transfusion (DST)

DA liver allografts were orthotopically transplanted into LEW recipients according to the method described by Kamada et al[17]. The cold ischemia time was <60 min and unhepatic time was <14 min. Transplanted animals were monitored on a daily basis. For the induction of tolerance, 1 ml of heparinized DA blood was administered (i.v.) to naïve LEW rats (125 g∼149 g) once a week for 4 weeks prior to LTx as previously described[18].

### Antibodies and Reagents and Analysis

Pacific Blue conjugated mouse anti rat CD4 antibody (W3/25) was obtained from AbD Serotec (Raleigh, NC). PerCP conjugated mouse anti-rat CD8a (OX-8), FITC conjugated mouse anti-rat CD25 (OX-39), FITC conjugated mouse anti-rat CD45RC (OX-22), PE conjugated mouse anti-rat CD45RC (OX-22) and APC conjugated mouse anti-rat CD3 (1F4) antibodies were purchased from BD Bioscience (San Jose, CA). PE anti-mouse/rat Foxp3 (FJK-16s) was from eBioscience (San Diego, CA). Splenocytes (1×10^6^ cells) or lymph nodes (LNs) surrounding the portal and splenic veins were stained with indicated antibodies and analyzed using flow cytometer (FACSVantage SE, BD Bioscience, San Jose, CA). For intracellular staining of Foxp3, cells stained with surface marker antibodies were fixed, permeabilized and incubated with PE conjugated anti-mouse/rat Foxp3 according to the manufacturer's protocol. Corresponding isotype-matched control mAbs were used in all flow cytometric staining procedures. All flowcytometric analysis was done using FlowJo software (Tree Star Inc. San Diego CA, ver7.2.5).

### T-Cell Isolation

Spleens were obtained from naïve LEW rats or LEW rats subjected to 1 to 4 weekly injections of DST alone (DSTx1 to DSTx4) or 4 weekly injections of DST in combination with LTx (DSTx4+LTx). Single cell suspensions were prepared and MHC class II^+^ cells were depleted using MACS Rat MHC-II MicroBeads (Miltenyi Biotec, Auburn, CA) according to the manufacturer's protocol to yield a lymphocyte population highly enriched (>95%) for CD3^+^ T-cells. Positive or negative selection for CD4^+^ and/or CD8^+^ cells were performed using MACS Rat CD4 and/or CD8 Microbeads. Cells stained for CD4 and CD45RC or CD25 mAb were sorted using the FACSAria Cell-Sorting System (BD Bioscience, San Jose, CA). Each population was determined to be >98% pure by flow cytometry.

### Mixed Lymphocyte Reaction (MLR)

Unfractionated T-cells, CD4^+^ T-cells or CD8^+^ T-cells (1×10^5^cells) from LEW rats were cultured in triplicate in U-bottom 96 well plates with 2×10^5^ irradiated (2,000 rad) DA splenocytes for 5 days at 37°C in complete RPMI-1640 as described previously[19]. For suppression assays, 1×10^5^ CD4^+^ T-cells or CD8^+^ T-cells from naïve or treated LEW rats were added to the standard MLR assay (using naïve LEW T-cells as responder cells) as described above. One μCi of [^3^H]-thymidine was added to each well for the last 18 hr incubation and [^3^H]-thymidine incorporations were quantified.

### Quantitative mRNA Cytokine Determinations

Real time PCR (RT-PCR) was used to quantify the expression of IL-10, TGFβ and IFNγ using the Applied Biosystems 7300 Real-Time PCR System and Power SYBR green Master Mix (Foster City, CA). HPRT served as internal control. All expression levels of the target genes were normalized to the housekeeping gene HRPT.

### Adoptive Transfer Studies

Naïve LEW rats were lightly-irradiated (100 rad) and varying numbers of whole splenocytes or indicated T-cell populations obtained from naïve, DSTx4 alone or DSTx4+LTx rats were injected (iv.) immediately following irradiation. Twenty four hours following adoptive transfer, DA livers were transplanted into the LEW recipients.

### Statistics

Mann-Whitney U tests were performed for comparison of means. Kaplan-Meier survival graphs were made and Log rank comparison of the groups was used to calculate p values. Statistical level of significance was defined as p<0.05.

## Results

### Allograft Tolerance Is Maintained for 30 but Not 100 Days Following DST Treatment

**Figure 1:**
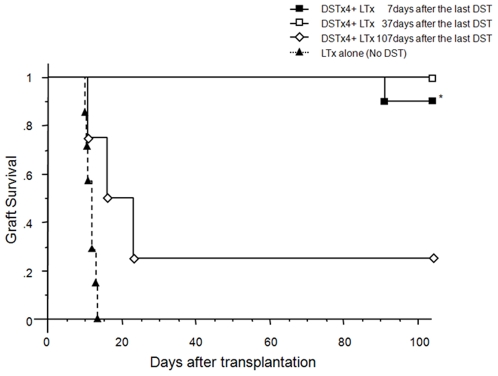
Donor specific blood transfusion (DST) induces tolerance to liver allografts. **LEW rats were pretreated with 1 ml of heparinized DA blood (DST) once per week for 4 weeks prior to DA liver transplantation (LTx).** LTx was performed 7 days following last DST treatment (DSTx4+LTx 7days; black squares, n = 10). DSTx4+LTx 37days (white squares, n = 5) and DSTx4+LTx 107days (white diamonds, n = 4) represent LEW rats pretreated once per week for 4 weeks with DST with LTx performed at 37 or 107 days following last DST treatment. Some animals did not receive DST but did receive LTx (black triangles, n = 7). *P<0.05 compared with LTx alone.

We have previously reported that DST induces tolerance to DA liver allografts and long term survival of LEW recipients[18]. In these previous studies we found that weekly DST treatments increased survival from 0% for rats that did not receive DST to 25%, 50% and 88% for recipients that received DST once per week for 1, 2, or 4 weeks prior to LTx respectively[18]. In the current study, we extend these original findings by demonstrating that tolerance induced by 4 weekly treatments of DA donor blood (DSTx4) is maintained even when LTx was delayed for more than one month (37 days) following the last DST treatment (Fig. 1). However, the tolerogenic effects of DSTx4 were lost when LTx was delayed >100 days following the last DST treatment (Fig. 1). It should be noted that tolerance to liver allografts in this strain combination is donor/antigen-specific as rat blood transfusion with a different genetically different strain of rat (e.g. Brown Norway rat) does *not* prevent DA allograft rejection and conversely, DA blood transfusion doe not induce tolerance to liver allografts obtained from a genetically different rat (e.g. PVG) 2when transplanted into LEW rats[18].

### DST Alone or in Combination with LTx Affects Alloantigen-Induced CD4^+^ T-Cell Activation In Vitro

**Figure 2:**
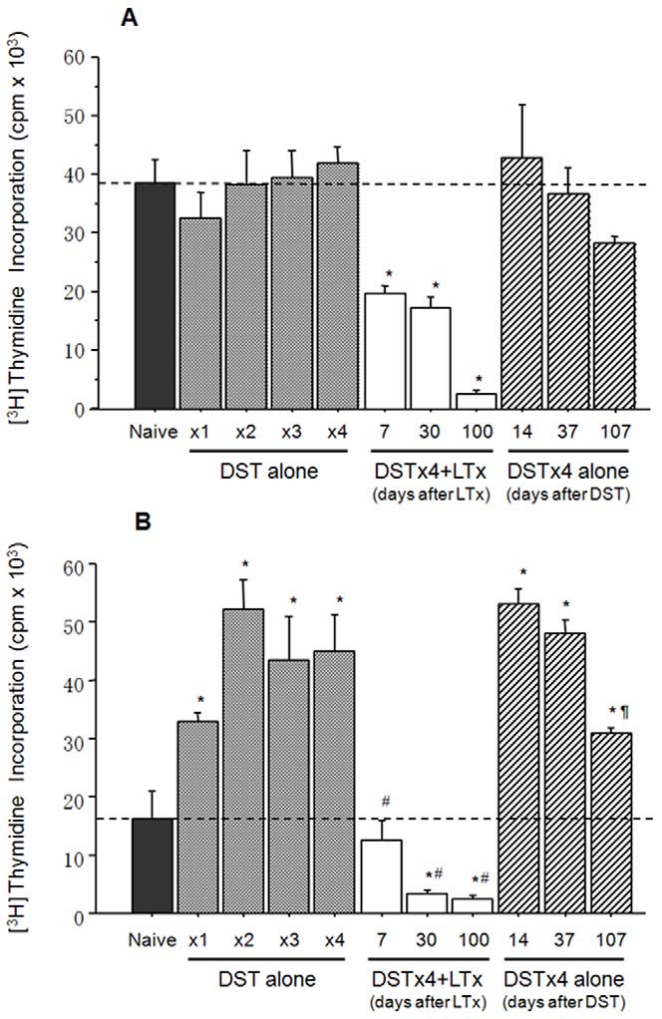
Alloreactive responses of T-cells obtained from LEW rats subjected to DST alone or DSTx4+LTx *in vitro*. (A) **T-cells** (1×10^5^) from LEW rats subjected to one or more weekly DST treatments with or without LTx were incubated with irradiated DA splenocytes (2×10^5^) for 5 days to induce T-cell proliferation. The dotted line shows the proliferation of T-cells from naïve LEW rats. DSTx4 alone at 14, 37 and 107 days represent alloreactive responses of T-cells obtained from LEW rats pretreated with 4 weekly DST treatments and then harvested at 14, 37 and 107 days following the last DST treatment (no LTx). *P<0.05 compared with proliferation of T-cells from Naïve LEW rat. (B) **CD4^+^ T-cells** (1×10^5^) from LEW rats subjected to one or more weekly DST treatments with or without LTx were incubated with irradiated DA splenocytes as described above. The dotted line shows the proliferation of CD4^+^ T-cells from naïve LEW rats. *P<0.05 compared with Naïve LEW rat. ^#^P<0.05 compared with DSTx4. ^¶^P<0.05 compared with DSTx4 alone at 14 d. Data represent mean±SD from at least three independent experiments.

As a first attempt to determine whether DST alone or in combination with LTx affects alloreactive responses of T-cells, we performed a series of MLR experiments *in vitro*. We found that alloantigen (DA)-induced activation of T-cells obtained from LEW rats pretreated at different times with DST alone was very similar to that of naïve T-cells (Fig. 2A). Alloantigen-induced activation of T-cells obtained from LEW animals subjected to DSTx4+LTx was significantly decreased in a time-dependent manner (Fig. 2A). This suppression of alloantigen-induced proliferation appeared to be dependent upon the combination of both DST and LTx as T-cells obtained from rats at various days following DSTx4 alone maintained their ability to respond to irradiated DA splenocytes (Fig. 2A). We found that the CD4^+^/CD8^+^ ratio (70/30) in these responder T-cells did not change throughout this experiment (data not shown).

To ascertain which T-cell subset was responsible for the suppressive effect observed in Figure 2A, we performed the same types of experiments as described above with CD4^+^ or CD8^+^ T-cells obtained from the different groups of rats. Interestingly, we found that alloantigen-induced proliferation of CD4^+^ T-cells obtained from rats that were subjected to multiple weekly DST increased in a dose-dependent fashion to more than 2–3 fold when compared to naïve CD4^+^ T-cells (Fig. 2B). CD4^+^ T-cells obtained from rats subjected to DSTx4+LTx were much less responsive to alloantigen-induced proliferation such that the proliferative activity of CD4^+^ T-cells obtained from LEW animals at 30 and 100 days following LTx was dramatically and significantly decreased as compared to naïve CD4^+^ T-cells (Fig. 2B). As with unfractionated T-cells, this decreased proliferative activity of CD4^+^ T-cells appeared to be dependent upon the presence of both DST and LTx as CD4^+^ T-cells obtained from rats at 14 and 37 days following DSTx4 alone maintained their hyper-proliferative state in response to alloantigens. However, CD4^+^ T-cells obtained from rats at 107 days following DSTx4 alone exhibited only 50% the proliferative response of cells obtained from rats at 14 days post DST treatment (Fig. 2B). Finally, we found that CD8^+^ T-cells obtained from naïve LEW rats or LEW animals treated with DST alone or in combination with LTx responded to irradiated DA splenocytes with <10% the proliferative response of CD4^+^ T-cells (data not shown).

### DST Alone or in Combination with LTx Affects the Regulatory Properties of CD4^+^ or CD8^+^ T-Cells In Vitro

**Figure 3:**
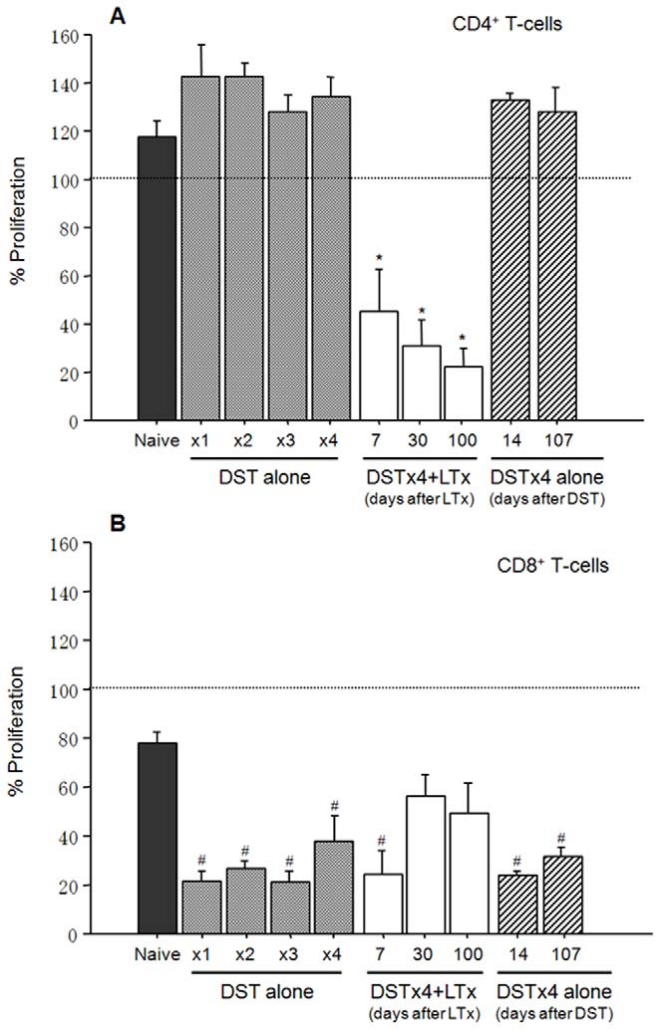
Suppressive activity of CD4^+^ or CD8^+^ T-cells from LEW rats subjected to DST alone or DSTx4+LTx *in vitro*. (A) CD4^+^ T-cells (1×10^5^) from rats subjected to one or more weekly DST treatments with or without LTx were incubated with donor (DA) irradiated splenocytes (2×10^5^) in the presence of naïve LEW T-cells (1×10^5^) as described above. The dotted line shows the proliferation of T-cells from naïve LEW rats (designated as 100%). DSTx4 alone at 14 and 107 days represent suppressive activity of CD4^+^ T-cells obtained from LEW rats pretreated with 4 weekly DST treatments and then harvested at 14 and 107 days following the last DST treatment. (B) CD8^+^ T-cells obtained from rats subjected to one or more weekly DST treatments with or without LTx were incubated with donor (DA) irradiated splenocytes (2×10^5^) in the presence of naïve LEW T-cells (1×10^5^) as described above. The dotted line shows the proliferation of T-cells from naïve LEW rats (designated as 100%). DSTx4 alone at 14 and 107 days represent suppressive activity of CD8^+^ T-cells obtained from LEW rats pretreated with 4 weekly DST treatments and then harvested at 14 and 107 days following the last DST treatment. Combined results are shown as mean±SE. * and ^#^ P<0.05 compared with either CD4^+^ T-cells or CD8^+^ T-cells from naïve LEW rat.

We next investigated the ability of CD4^+^ or CD8^+^ T-cells obtained from the different groups of rats to suppress alloantigen-induced activation of naïve LEW T-cells *in vitro*. We found that CD4^+^ T cells obtained from rats treated with one or multiple DST were not able to inhibit alloantigen-induced T-cell proliferation (Fig. 3A). However, when DSTx4 was combined with LTx, CD4^+^ T-cells obtained from these animals at 7, 30 and 100 days post LTx acquired potent suppressive activity (Fig. 3A). Again this effect appeared to be dependent upon the presence of donor antigens (ie allograft), as CD4^+^ T-cells from LEW rats treated with DSTx4 alone at 14 or 107 days post DST were unable to suppress alloantigen-induced proliferation of naïve LEW T-cells (Fig. 3A). In contrast to CD4^+^ T-cells, CD8^+^ T-cells obtained from rats treated with DST alone displayed significant suppressive activity under the same experimental conditions (Fig. 3B). This suppressive activity was abrogated at 30 or 100 days after LTx. CD8^+^ T-cells maintained their suppressive activity for >100 days following DST treatment alone (Fig. 3B). This suppressive activity was donor antigen specific because CD8^+^ T-cells did not suppress naïve T-cell proliferation induced by irradiated PVG splenocytes (third party) and CD8^+^ T cells from rats receiving PVG blood transfusion were not able to inhibit naive T cell proliferation induced by irradiated DA splenocytes (data not shown).

### Time-Dependent Increase in CD4^+^T-Cells Expressing Foxp3 and CD25 in DSTx4+LTx Recipients

**Figure 4:**
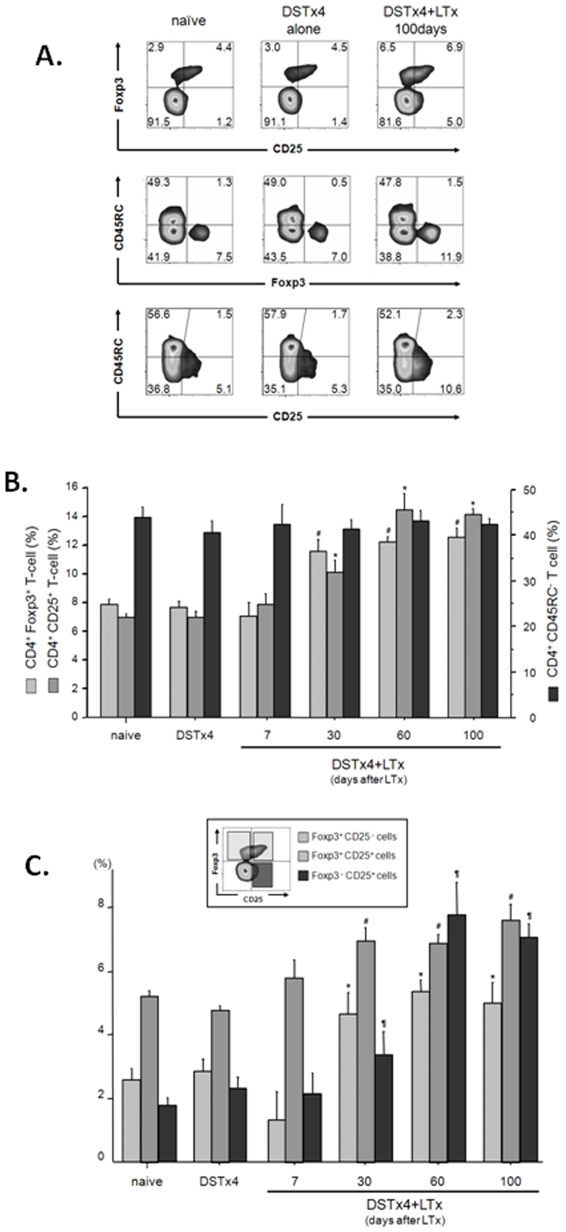
Alterations in different CD4^+^ populations in spleens obtained from LEW rats subjected to DST in the absence or presence of LTx. (A) Representative flow cytometric analysis of splenocytes harvested from either naïve, DSTx4 alone or DSTx4+LTx rats 100days after LTx. Cells are gated on CD3^+^ CD4^+^ T-cells. Isotype-matched control antibodies were used to set all gates. (B) Percentages of Foxp3^+^, CD25^+^ or CD45RC^−^ T-cells within the CD4^+^ T-cell population in spleens from naive, DST treated or DSTx4+LTx rats. (C) Percentages of Foxp3^+^CD25^−^, Foxp3^+^CD25^+^ or Foxp3^−^CD25^+^ T-cells within the CD4^+^ T-cell population in spleens from naïve, DST treated or DSTx4+LTx rats. The results are expressed as mean±SE from at least three individual experiments for each group. * ^# ¶^ P<0.05 compared with corresponding population in naïve group.

To determine whether DST alone or in combination with LTx induces the generation of Foxp3-expression regulatory T-cells (Tregs), we evaluated whether DST treatment in absence or presence of LTx affects the percentages of different T-cell subsets thought to possess regulatory function *in vivo*. We found that DST treatment alone did not alter expression of Foxp3, CD25 or CD45RC on CD4^+^ T-cells obtained from spleens of these rats (Fig. 4A and 4B). In addition, no alterations in expression of these markers were observed when spleens were obtained from rats at 7days following LTx (Fig. 4B). We did observe significant increases in Foxp3 and CD25 but not CD45RC expression on CD4+ T-cells obtained from rats at 30, 60 and 100 days following DSTx4+LTx compared to those from naïve rats (Fig. 4B). Interestingly, both CD25 and Foxp3 expression were increased in the CD45RC^−^ population from long term survivors (DSTx4+LTx >100day survival) despite the fact that percentages of CD4^+^CD45RC^−^ T-cells remained unchanged when compared to naïve T-cells or T-cells obtained from DSTx4 treated rats (Fig. 4A and 4B). In addition, both Foxp3^+^CD25^−^ and Foxp3^+^CD25^+^ cells significantly increased in a time-dependent manner in the CD4^+^ T-cell population obtained at 30 days following LTx (Fig. 4C). Although CD4^+^CD25^+^ T-cells also increased, Foxp3^−^ T-cells increased more than did Foxp3^+^ T-cells in CD4^+^CD25^+^ population. In fact, we found that only 50% of CD4^+^CD25^+^ T cells expressed Foxp3^+^ in the long term survivors (Fig. 4C). No alterations in expression of CD25, CD45RC and/or Foxp3 were noted in CD8^+^ T-cells and similar data were obtained for T-cells obtained from LNs surrounding the portal vein (data not shown).

### Foxp3^+^CD25^+^ T-Cells from Long Term Survivors Express More TGFβ mRNA than Do Foxp3^−^CD25^+^ T-Cells

**Figure 5:**
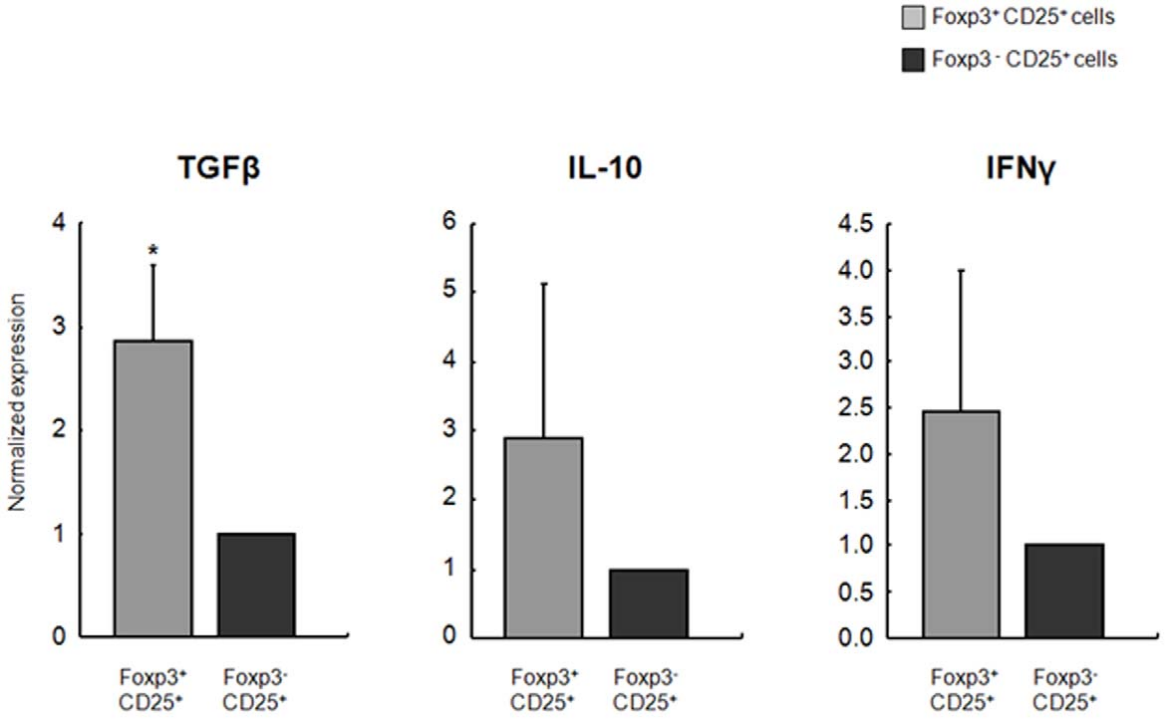
Cytokine mRNA Expression by Foxp3^+^CD25^+^ and Foxp3^−^CD25^+^T-cells from long term survivors. Message levels of TGFβ, IL-10 and IFNγ in CD4^+^Foxp3^+^CD25^+^ T-cells obtained from spleens of long term survivors (DSTx4+LTx rats; survival>100 days) were quantified and compared to those expressed by CD4^+^Foxp3^−^CD25^+^ T-cells obtained from these same animals. Quantitative Real-time (RT)-PCR was used to quantify cytokine mRNA expression in each population. HRPT was used as an endogenous control to normalize each sample. Data represent mean±SD. * P<0.05 compared with Foxp3^−^CD25^+^ T-cells. N = 5 for each group.

Message levels of the different cytokines from CD4^+^Foxp3^+^CD25^+^ T-cells obtained from spleens of long term survivors (DSTx4+LTx rats; survival>100 days) were quantified and compared to those expressed by CD4^+^Foxp3^−^CD25^+^ T-cells from these same animals. We found that although TGFβ, IL-10 and IFN-γ mRNA expression was increased in Foxp3^+^ vs. Fop3^−^ T-cells, only TGFβ expression was significantly increased (Fig. 5).

### Adoptive Transfer of CD4^+^ T-Cells Obtained from Rats Subjected to DST Alone Prolongs Survival of Rat Liver Allografts

**Figure 6:**
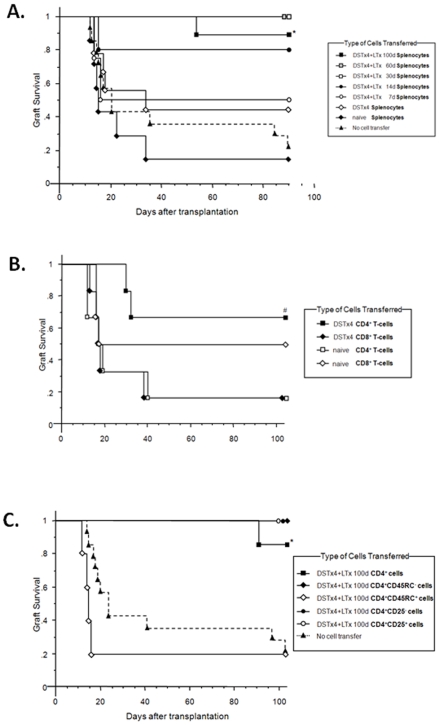
Allograft survival following adoptive transfer of splenocytes or indicated T-cell subsets obtained from different treatment groups. (A) Adoptive transfer of whole splenocytes from DSTx4+LTx rats induces tolerance to liver allografts in naïve LEW recipients in a time-dependent manner. Splenocytes (250×10^6^ cells) obtained from naïve (black diamonds, n = 7), DSTx4 (white diamonds,n = 9), DSTx4+LTx at 7 d post LTx (white circles, n = 4), DSTx4+LTx at 14 d post LTx (black circles, n = 5), DSTx4+LTx at 30 d post LTx (white squares, n = 4), DSTx4+LTx at 60 d post LTx (hash squares, n = 4) or DSTx4+LTx at 100 d post LTx (black squares, n = 9) were injected into lightly-irradiated (100rad) LEW rats one day before LTx. Control rats received 100 rad irradiation and received LTx but no cell transfer (black triangles, n = 14). *P<0.05 compared with no cell transfer. (B) Adoptive transfer of CD4^+^ but not CD8^+^ T-cells obtained from DST-primed LEW rats induces tolerance to liver allografts in naive LEW recipients. CD4^+^ (white squares, n = 6) or CD8^+^ (white diamonds, n = 6) T-cells (50×10^6^ cells) obtained from spleens of naïve rats and CD4^+^ (black squares, n = 6) or CD8^+^ (black diamonds, n = 6) T-cells (50×10^6^ cells) obtained from spleens of DSTx4 rats were injected into lightly-irradiated (100 rad) LEW rats one day before LTx. ^#^P = 0.05 compared with naïve CD4^+^ (white squares). (C) Adoptive transfer of CD4^+^CD45RC^−^, CD4^+^CD25^+^ or CD4^+^CD25^−^ T-cells induces tolerance to liver allografts in naïve LEW recipients. CD4^+^ T-cells (50×10^6^ cells; (black squares, n = 7); CD4^+^CD45RC^−^T-cells(20×10^6^ cells; black diamonds, n = 5); CD4^+^CD45RC^+^ T-cells (20×10^6^ cells; white diamonds,n = 5); CD4^+^CD25^−^ T-cells (40×10^6^ cells; black circles, n = 5); and CD4^+^CD25^+^ T-cells (10×10^6^ cells, white circles, n = 5) from DSTx4+LTx at 100days post LTx were transferred into lightly irradiated LEW rats one day prior to LTx. *P<0.05 compared with no cell transfer (black triangles).

We next wished to determine whether adoptive transfer of whole splenocytes obtained from the various groups of rats into a second set of naïve lightly irradiated (100 rad) LEW recipients 24 hrs prior to LTx induced long term survival as described previously for heart, kidney or skin allografts[12], [16], [19], [20]. Adoptive transfer of splenocytes from *naïve* LEW donors resulted in a 100 day survival of only 15% of recipients that received LTx (Fig. 6A). Transfer of splenocytes from LEW rats treated with DSTx4 alone increased survival to 40% however this increase was not statistically significant. One hundred day survival was increased to 90–100% when splenocytes from long term survivors were transferred into lightly irradiated LEW recipients prior to LTx. Furthermore, we observed a time-dependent increase in survival depending upon *when* the splenocytes were isolated from DSTx4+LTx rats. We found that adoptive transfer of splenocytes obtained from DSTx4+LTx rats at 7, 14, 30 and 60 days following LTx increased survival from 15% in rats that received naïve splenocytes to 50, 80, 100 and 100%, respectively at 100 days post LTx (Fig. 6A).

Although whole splenocytes from DST-alone rats did not increase significantly survival of a second set of recipients, our *in vitro* data suggested that DST alone induces the production of CD8^+^ T-cells that suppresses alloantigen-induced proliferation of naïve T-cells (Fig. 3B) and promotes a hyper-proliferative response of CD4^+^ T-cells to alloantigens (Fig. 2B). Therefore, we next investigated whether adoptive transfer of T-cells enriched for either CD8^+^ or CD4^+^ cells obtained from rats subjected to DSTx4 alone into naïve LEW animals prior to receiving LTx affected allograft survival *in vivo*. Unexpectedly, we found that adoptive transfer of CD8^+^ T-cells obtained from DSTx4 treated rats to lightly irradiated (100 rad) LEW recipients did not prolong allograft survival whereas adoptive transfer of CD4^+^ T-cells from DSTx4-treated rats significantly increased allograft survival compared to the rats that received *naïve* CD4^+^ or CD8^+^ T-cells (Fig. 6B). Moreover, we observed similar increases in Foxp3 and CD25 expression on CD4^+^T-cells obtained from the second set of recipients that received CD4^+^ T-cells from rats subjected DSTx4 alone when analyzed by flow cytometry(100days after LTx; data not shown).

### Alloantigen Specific Tregs Induced by DST+LTx Are CD4^+^CD45RC^−^Foxp3^+^ T-Cells

For the next series of experiments, we enriched splenocytes obtained from long term survivors (DSTx4+LTx; >100day survival) for CD4^+^ T-cells and transferred these cells into lightly irradiated LEW recipients 24 hr prior to LTx. We found that the 100 day survival increased significantly from 21% in irradiated LEW rats that received LTx but no T-cells to almost 90% for rats that received CD4^+^ T-cells from long term survivors (Fig. 6C). Because most, if not all of the Foxp3 expressing CD4^+^ T-cells are found in the CD4^+^CD45RC^−^ T-cell population of long term survivors, we sorted the DSTx4+LTx splenocytes into the CD4^+^CD45RC^+^and CD4^+^CD45RC^−^ populations and transferred these cells to a second set of naïve recipients. Adoptive transfer of CD4^+^CD45RC^−^ T-cells dramatically increased the 100 day survival of the recipients to 100% whereas transfer of CD4^+^CD45RC^+^ T-cells did not increase survival when compared to control LTx rats that did not receive T-cells (Fig. 6C). In addition, adoptive transfer of CD4^+^CD25^+^ or CD4^+^CD25^−^ cells from DSTx4+LTx donor rats increased survival in both groups, suggesting the presence of Tregs in both the CD25^+^ and CD25^−^ T-cell populations (Fig. 6C).

## Discussion

Data presented in the current study demonstrate the novel finding that adoptive transfer of DST-primed *CD4^+^ T-cells* suppresses liver allograft rejection despite the fact that these T-cells express little or no Foxp3 and possess no MLR suppressive activity *in vitro*. These are somewhat surprising findings given the fact that previous studies have demonstrated that transfer of *whole splenocytes* from animals treated with DST alone is insufficient to induce long term survival of heart and kidney allografts[16], [20], [21]. Indeed, we did observe that adoptive transfer of *whole splenocytes* obtained from DST treated rats failed to significantly extend allograft survival when compared to rats that were injected with splenocytes from naïve LEW donors (Fig. 6A). However, when *CD4^+^* (but not *CD8^+^ T-cells*) were obtained from rats that had been pretreated with DSTx4 alone were transferred into naïve recipients 24 hrs prior to LTx, we observed a significant increase in survival of the liver allografts compared to LTx animals receiving naïve CD4^+^ T-cells (Fig. 6B). Our data coupled to data from other laboratories [22], [23] suggest that CD8^+^ T-cells may be required for the *DST-induced priming* of CD4^+^ T-cells but are not themselves the cellular mediators of DST-induced tolerance to tissue allografts. Currently, it is not clear why DST-primed whole splenocytes fail to induce tolerance whereas CD4^+^ T-cells enriched from DST-primed splenocytes do. It may be that one or more lymphocyte populations (ie CD8^+^ T-cells, B-cells) generated in DST-primed animals “interfere” with or suppress the development and/or regulatory function of DST-primed CD4^+^ T-cells. Indeed, when CD8^+^ T-cells are removed from unfractionated DST-primed T-cells, the remaining CD4^+^ T-cells respond to alloantigens with a 2–3 fold higher proliferative response (Fig. 2B) suggesting that CD8^+^ T-cells actively suppress alloantigen activation of CD4^+^ T-cells. This idea is supported by our data demonstrating that addition of DST-primed CD8^+^ T-cells potently suppressed alloantigen-induced activation of naïve T-cells *in vitro* (Fig. 3B). Obviously, the relationship between CD4^+^ and CD8^+^ T-cells during DST priming and maintenance periods is in need of further investigation. Another lymphocyte population that may play a role in regulating the development and/or function of DST-primed CD4^+^ Tregs *in vivo* is the B-cell. It is known for example that B-cells block Treg function thereby exacerbating Th1-mediated gut inflammation in a mouse model of Crohn's disease[24].

The mechanisms by which transfer of DST-primed CD4^+^ T-cells suppress allograft rejection *in vivo* have not been identified however these T-cells appear to represent a Treg precursor population that develops into fully functional Tregs in the presence of donor antigens. Closer examination of the phenotype and functional properties of these DST-primed CD4^+^ T-cells vs. those obtained from DSTx4+LTx clearly reveal 2 distinct populations of T-cells. For example, DST-primed CD4^+^ T-cells respond to alloantigens with a hyper-proliferative response whereas CD4^+^ T-cells obtained from DSTx4+LTx exhibit little or no alloantigen-induced proliferation *in vitro* (Fig. 2B). It may be that the rapid infiltration and proliferation of T-cells within the transplanted allografts following DST treatment as observed by others[21], [25], [26] represents alloantigen-induced activation of DST-primed CD4^+^ T-cells. A second set of observations that suggest DST-primed CD4^+^ T-cells are distinct from functional Tregs induced by DSTx4+LTx is that DST-primed T-cells express little or no Foxp3 and do not suppress alloantigen-induced activation of naïve T-cells *in vitro* whereas DSTx4+LTx CD4^+^ Tregs are CD4^+^CD45RC^−^Foxp3^+^ and dramatically suppress alloantigen-induced proliferation of naïve T-cells *in vitro* (Fig. 3A and 4). Previous work from our laboratory have suggest the generation of fully functional Tregs is both dose and time dependent with respect to DST administration[18]. Data obtained in the current study extend these observations by demonstrating that the tolerance induced by DSTx4 is maintained even when LTx was delayed by 30 days following the last DST treatment. Furthrmore, we found that tolerace lost when LTx was delayed 100days post-DST suggesting that alloantigen-primed CD4^+^ T-cells have extended but finite lifetimes in the absence of continuous alloantigen exposure (Fig. 1). Although, the mechanisms by which continuous alloantigen exposure is required for DST-induced, long term tolerance have not been delineated, they may be related to the strength of proliferative response of CD4^+^ T-cells to donor antigen (Fig. 2B). Taken together, we propose that DST-primed, hyperproliferative CD4^+^ T-cells represent a precursor population that may ultimately develop into fully functional Tregs (CD4^+^CD45RC^−^Foxp3^+^) in the presence of alloantigens expressed by the liver graft (Fig. 2, 4 and 6A). Indeed, it may be that the sinusoidal endothelial cells play an important role in inducing tolerance to alloantigens.

Another novel finding we have made in the current study is the time-dependent increase in the suppressive activity of T-cells *in vitro* (Fig. 2) and *in vivo* (Fig. 6A). We dmeonstrate that T-cells (or splenocytes) isolated from DSTx4+LTx rats at 30, 60 and 100 days post LTx are much more suppressive against alloantigen-induced activation of T-cells as well as allograft rejection than were T-cells isolated at 7 or 14 days post LTx (Fig. 2 and 6A). This time-dependent increase in suppressive activity correlated well with increased frequency of Foxp3-expressing Tregs (Fig. 4B and 4C). These data contrast to those of Kitade and coworkers who suggested that functional Tregs were present very early following DST+heart allograft transplantation[16]. This conclusion was based upon the observation that splenocytes, obtained at 5 days following heart allograft transplantation (and preoperative DST), could transfer tolerance to naïve recipients receiving heart allografts[16]. However, it should be pointed out that >10 times the number of splenocytes were required to suppress allograft rejection in these specific studies when compared to the numbers of splenocytes that were required for tolerance when obtained at later times (14 or 30 days) following heart allograft transplantation[16]. Furthermore, adoptive transfer of splenocytes obtained at 5 days post transplantation induced less than 40% allograft survival at 90 days following surgery[16]. Taken together, our data suggest that the numbers of allo-antigen specific Tregs are small and/or their suppressive function has not fully developed during the first week or two following allograft transplantation. We propose that DST-primed T-cells require several weeks to mature into fully functional Tregs and that this process requires the presence of alloantigens (e.g. allograft).

Finally, we present the novel observation that virtually all of the Foxp3-expressing Tregs generated by DSTx4+LTx reside within the CD4^+^CD45RC^−^ population and that these Tregs suppress alloantigen-induced activation *in vitro* as well as induce long term tolerance to liver allografts *in vivo* (Fig. 4A and 6C).

Although we did observe significant numbers of Foxp3-expressing Tregs in the CD4^+^CD25^+^ population, we also identified an equal number of CD^+^Foxp3^+^ Tregs present in the CD4^+^CD25^−^ subset. These data suggest that the CD45RC^−^ population rather than CD25^+^ subset of CD4^+^ T-cells contains the largest percentages of functional Tregs in this animal model.

In summary, our data suggest that DST administration, in the absence of allograft transplantation, induces the formation of CD4^+^ T-cells that are not Tregs themselves but give rise directly or indirectly to fully functional CD4^+^CD45RC^−^Foxp3^+^Tregs following liver allograft transplantation.
